# Performance Evaluation of a Smart Bed Technology against Polysomnography

**DOI:** 10.3390/s22072605

**Published:** 2022-03-29

**Authors:** Farzad Siyahjani, Gary Garcia Molina, Shawn Barr, Faisal Mushtaq

**Affiliations:** Sleep Number® Labs, San Jose, CA 95113, USA; gary.garciamolina@sleepnumber.com (G.G.M.); shawn.barr@sleepnumber.com (S.B.); faisal.mushtaq@sleepnumber.com (F.M.)

**Keywords:** ballistocardiography, breathing rate, heart rate

## Abstract

The Sleep Number smart bed uses embedded ballistocardiography, together with network connectivity, signal processing, and machine learning, to detect heart rate (HR), breathing rate (BR), and sleep vs. wake states. This study evaluated the performance of the smart bed relative to polysomnography (PSG) in estimating epoch-by-epoch HR, BR, sleep vs. wake, mean overnight HR and BR, and summary sleep variables. Forty-five participants (aged 22–64 years; 55% women) slept one night on the smart bed with standard PSG. Smart bed data were compared to PSG by Bland–Altman analysis and Pearson correlation for epoch-by-epoch HR and epoch-by-epoch BR. Agreement in sleep vs. wake classification was quantified using Cohen’s kappa, ROC analysis, sensitivity, specificity, accuracy, and precision. Epoch-by-epoch HR and BR were highly correlated with PSG (HR: r = 0.81, |bias| = 0.23 beats/min; BR: r = 0.71, |bias| = 0.08 breaths/min), as were estimations of mean overnight HR and BR (HR: r = 0.94, |bias| = 0.15 beats/min; BR: r = 0.96, |bias| = 0.09 breaths/min). Calculated agreement for sleep vs. wake detection included kappa (prevalence and bias-adjusted) = 0.74 ± 0.11, AUC = 0.86, sensitivity = 0.94 ± 0.05, specificity = 0.48 ± 0.18, accuracy = 0.86 ± 0.11, and precision = 0.90 ± 0.06. For all-night summary variables, agreement was moderate to strong. Overall, the findings suggest that the Sleep Number smart bed may provide reliable metrics to unobtrusively characterize human sleep under real life-conditions.

## 1. Introduction

Increased awareness of the importance of sleep, together with the advancement of consumer electronics technology, has resulted in the emergence of consumer sleep-tracking devices [1] that combine outputs from sensors with apps [2,3,4] and proprietary software [3]. These products use one or a combination of physiological signals to evaluate sleep: motion and movement (e.g., using wrist-worn accelerometers [3] or bedside radar detection [5]), brain activity signals (e.g., using dry-electrode headbands [6,7]), heart rate (HR; e.g., using chest belts [1]), breathing patterns (e.g., using chest patches [1,3]), cardiac activity (e.g., using optical pulse sensors [3]), and skin temperature (e.g., using wearable thermometers [1]).

The Sleep Number smart bed evaluates the sleeper’s bed presence, body movements, heartbeat, and breathing patterns in real time to determine the duration and quality of sleep. Similar to bed surfaces in sleep laboratories or specialized medical care units [8,9,10,11] and to under-mattress monitoring devices used by consumers [8], the smart bed sensing technology uses high-resolution full-body ballistocardiography (BCG) readings from an inflatable air bladder within the bed mattress, underlying the entire body of a single sleeper. The use of BCG as a passive-sensing technology enables the measurement of motion, position changes, breathing, and small movements within the body such as those generated by the ejection of blood with each heartbeat [12,13]. The smart bed sensors automatically begin data collection as soon as the user enters the bed and stop once the user leaves. Smart bed users receive information about estimated sleep timing, duration, and quality via a consumer smart phone app on a daily basis. Processed data are sent to the cloud and stored for further analysis.

Most sleep-tracking products require the sleeper to carry, wear, or attach a device, monitor battery charge, and/or manually mark “time in bed” to measure sleep behavior. Movement alone has limited specificity in tracking sleep patterns [14]. Newer sleep-tracking devices incorporate information from cardiovascular signals [15,16] in addition to triaxial accelerometers, which have contributed to increased accuracy in sleep/wake classification [17,18]. Despite these improvements, sleep-tracking devices have some disadvantages, including inaccuracy due to single location of signal sensing, inconvenience, susceptibility to environmental disturbances (e.g., external vibrations), device misplacement or displacement during sleep, sensor damage, limited battery life, and the need for manual intervention to mark sleep-related events such as sleep intent or bedtime. For these reasons, 34% of users abandon their device within 9 weeks [19]. In addition, a limited number of wearables and nearables have been clinically validated against standard sleep monitoring technologies [20].

Polysomnography (PSG) is the gold standard for sleep monitoring in clinical, field, and home settings. PSG includes an electroencephalogram, electrocardiogram (ECG), electromyogram, electrooculogram, and the measurement of breathing signals. PSG also represents the gold standard by which consumer sleep technologies are validated. Although its use in longitudinally monitoring sleep in real-life (ecologically valid) conditions is possible, it is expensive, time consuming, and imposes a high burden on the user.

The smart bed platform offers a potential alternative for tracking sleep-dependent cardiorespiratory function and sleep versus wake states without the need for a wearable device or PSG. As a first step to validating the use of the smart bed platform in sleep research, the present study performed three analyses evaluating the performance of the smart bed against PSG. First, the smart bed was assessed for its ability to measure all-night HR and breathing rate (BR). HR and BR data generated by the smart bed were compared to HR and BR data measured by the PSG system’s ECG and flow channels, respectively. Comparisons were made at the epoch-by-epoch level using the Bland–Altman method and Pearson correlation. Second, epoch-by-epoch comparisons were made to assess the smart bed’s sensitivity for detecting sleep, specificity of detecting wake, accuracy, precision, Cohen’s kappa value, prevalence and bias-adjusted kappa, and area under the receiver operating characteristic curve (AUC). Finally, all-night summary metrics including sleep efficiency (SE), sleep onset latency (SOL), total sleep time (TST), and wake after sleep onset (WASO) were compared between the smart bed estimates relative to PSG using Bland–Altman metrics.

## 2. Materials and Methods

### 2.1. Participants

Forty-five adults who were self-described ‘normal sleepers’ volunteered for the study and participated in an overnight laboratory PSG at the Lakeland Sleep Center, Minneapolis, MN, between February 2016 and March 2016. Demographic data are provided in Table 1. All participants were over 18 years of age and provided written consent to participate in product research with the Sleep Number Corporation. To be included in the study, each participant was required to spend one full night in the sleep laboratory between 21 February 2016, and 31 March 2016, and to provide their body mass index (BMI). No exclusion criteria were applied for participant recruitment, and the participants were free of medical, neurological, or psychiatric illnesses based on a health status questionnaire. The study was designed for product research by the Sleep Number Corporation, and it was reviewed by the University of Chicago Institutional Review Board (IRB) for the analysis of de-identified smart bed and PSG data. The IRB gave the study exempt status.

Before participant recruitment, the target sample size was calculated using confidence interval analysis for correlations, as outlined by Moinester and Gottfried [21]. For this analysis, confidence intervals for the correlations between smart bed-measured HR or BR versus PSG were used. This approach showed that the sample size needed for a correlation of 0.85 and confidence interval width of 0.1 was 36. This figure was rounded up to 40, which became the target threshold for participant recruitment. As described in the Participants section, no exclusion criteria were applied, and there was no bias in the selection of participants; hence, all 45 participants were included for further analysis.

### 2.2. Protocol

Participants underwent one night of simultaneous PSG and smart bed recordings in a laboratory setting. All studies were performed while the participants were sleeping alone on the same model of queen-size (60 in × 80 in) smart bed. Smart bed data were collected every second and uploaded in real time to a secure cloud database. At the end of each recording, PSG data from lights out to lights on were extracted and uploaded to a secure database for further processing. Timing of lights off and lights on were based on each participant’s usual sleep schedule; thus, time in bed varied from one participant to the next. All participants’ data were anonymized.

### 2.3. Overnight Polysomnography

A standard montage was used for the PSG recordings, including 6-lead electroencephalogram signals (F3-A2, C3-A2, O1-A2, F4-A1, C4-A1, O2-A1), electrooculogram, electromyogram, electrocardiogram, thoraco-abdominal effort via respiratory impedance plethysmography, oronasal airflow using thermistor and nasal pressure base flow measurement, SpO2 with pulse oximetry, and body position (SomnoStar Sleep System, Vyaire Medical, Mettawa, IL, USA). PSG sleep stages were independently scored by three independent, registered sleep technicians based on the American Academy of Sleep Medicine guidelines, and the final sleep stage for each epoch was chosen as the stage scored by at least two of the three technicians. In the case of disagreement among the three scorers, a sleep-staging expert (G.G.M.) made the final determination. The resulting consensus scores were used in the evaluation of the smart bed sleep/wake detection.

### 2.4. Sleep Monitoring with Smart Bed Technology

Smart bed technology is built on an advanced Internet of Things [22] architecture that incorporates devices embedded with sensors, software, network connectivity, and other necessary electronics, enabling the collection and exchange of data in real-time. The bed’s embedded software employs advanced biomedical signal processing and machine-learning algorithms to determine physiological biomarkers. Processed data are sent to the cloud every second via WiFi, where they are stored and further analyzed to generate summary data for the night and sleep metrics. In the event of Wi-Fi or network failure, the device can store up to 4 h of data and send it once the connection is re-established.

**Figure 1:**
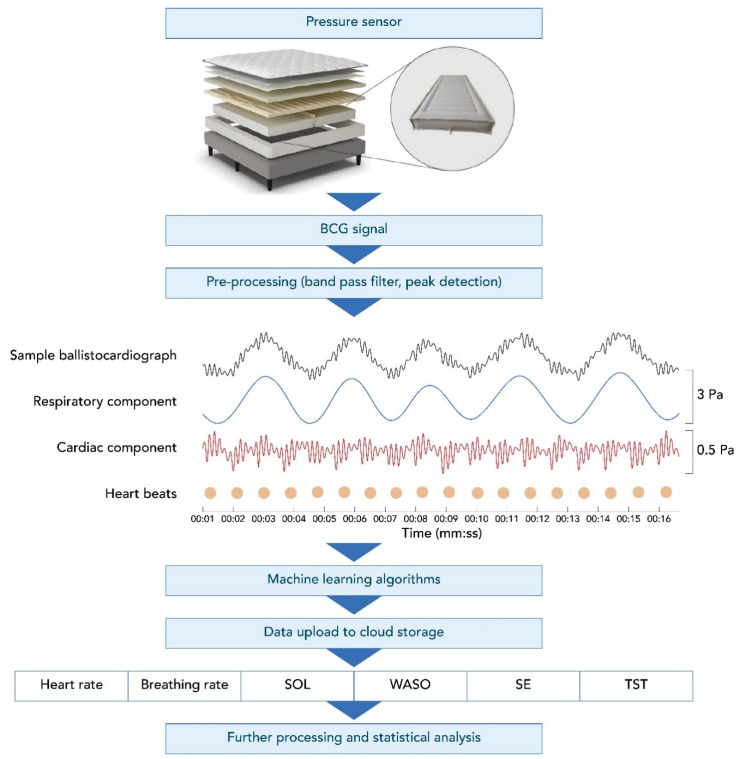
The smart bed setup, ballistocardiograph, and subsequent output. A BCG recording using the smart bed technology (black, top row of middle panel) during a motionless, 16 s recording segment decomposed into the respiratory pattern (blue, second row of middle panel) and heart activity (red, bottom row of middle panel). The dots denote detected individual heartbeats. Vertical axes are pressure readings in pascal units scaled for better visualization. BCG, ballistocardiography; SE, sleep efficiency; SOL, sleep onset latency; TST, total sleep time; WASO, wake after sleep onset.

The Sleep Number smart bed uses high-resolution, full-body BCG sensing that collects data at a rate of 1000 samples/second. Figure 1 shows an example of a BCG recording from the smart bed during a motionless, 16 s-long, sleep segment. This figure illustrates the decomposed breathing and cardiac components of the BCG signal. The smart bed algorithms determine the instantaneous HR and BR, which can track changes in HR and BR over time, creating longitudinal profiles of HR and BR. The data captured by the smart bed detect changes in HR and BR as the body progresses from wakefulness through stages of sleep [23,24,25,26]. The smart bed’s deep neural network (DNN) was developed in a separate study on over 1000 h of synchronized high-resolution BCG and PSG data to score 30 s epochs as wake vs. sleep. The smart bed DNN was trained to map the cardiac and respiratory signals and motion data embedded within the high-resolution BCG signal to PSG-derived sleep/wake states. These states were determined for each PSG-defined epoch. The overall approach is similar to that of other DNNs that have successfully detected different sleep stages using PSG signals [27,28,29,30,31,32,33,34].

### 2.5. Data Curation and Cleaning and PSG-Smart Bed Synchronization

No data from any given participant were excluded due to the identification by PSG of an undiagnosed sleep disorder. Any epoch with PSG artifacts that led to missing HR or BR detection was excluded from the analysis. From the remaining epochs, any epoch for which the smart bed system did not produce a HR or BR reading was noted as a “not covered” epoch. All remaining epochs were included in the analysis. Details on epoch coverage are provided in Appendix A.

Smart bed data were synchronized with PSG data using the time stamps associated with the two processor clocks. However, an additional manual alignment step was performed to correct for any possible drift between the internal clocks of the PSG and smart bed systems. In this manual step, large-amplitude movements were used as the basis for determining the time shift between PSG and smart bed data collection, since these movements were clearly visible in both the PSG and smart bed outputs. Movement was detectable in both systems because the BCG sensor output from the smart bed was electronically forked, with one line serving as an input to the PSG via a DC channel.

**Figure 2:**
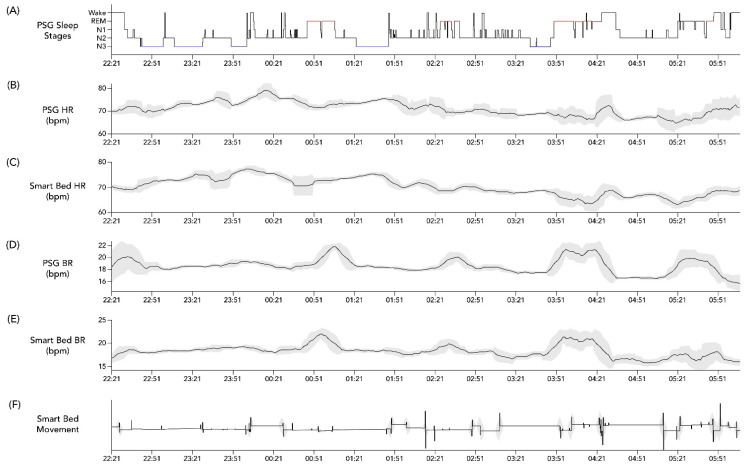
Example hypnogram from PSG and concurrent physiological data from PSG and the smart bed. Hypnogram data from a 36-year-old woman with BMI = 21.6 kg/m^2^ (**A**) and simultaneous profiles of PSG HR (**B**), smart bed HR (**C**), PSG BR (**D**), smart bed BR (**E**), and smart bed movement (**F**). Variability in overnight HR and BR was calculated at each time point as the standard deviation of values recorded over a running 15 min window (shaded grey bands). A 15 min smoothing window was applied to the HR and BR profiles to remove sporadic spikes and highlight the overall trends. BMI, body mass index; bpm, beats/breaths per minute; BR, breathing rate; HR, heart rate; PSG, polysomnography.

Lastly, an exploratory analysis using cross-correlation calculations was conducted on the overnight temporal profiles of HR and BR assessed from PSG as compared to the corresponding smart bed signals. After applying a 15 min smoothing window, these profiles were aligned to the PSG-based hypnogram to determine whether the expected physiological changes in HR and BR occurred in parallel in the PSG-based profile versus in the smart-bed-based profile. An individual example is illustrated in Figure 2. In the case of this representative subject, the cross correlation between PSG HR and smart bed HR was 0.6195 (*p* < 0.0001), and the cross correlation between PSG HR and smart bed HR was 0.490 (*p* < 0.0001), consistent with the visual concordance apparent in Figure 2. This exploratory analysis was used to identify conditions where dissociations between PSG and smart bed signals may be more likely to occur.

### 2.6. Epoch-by-Epoch Analysis

Following data alignment and exclusion of epoch with PSG artefact or/and with no coverage from the smart bed technology, epoch-by-epoch comparisons were made between the smart bed and PSG to assess the ability of the smart bed to detect HR and BR and consequently sleep/wake states. Epoch values for HR and BR were obtained by computing the mean of the instantaneous HR or BR values detected within each 30 s epoch.

### 2.7. Analysis of All-Night Summary Variables

All-night summary variables, including mean HR, mean BR, sleep onset latency (SOL), wake after sleep onset (WASO), total sleep time (TST), and sleep efficiency (SE) were compared between the smart bed and PSG. The reference BR was calculated from the PSG’s flow and effort signals. The channel with the dominant physiological pattern was visually examined and manually selected. Afterward, local maxima for every breathing cycle were detected using a peak detection algorithm. The reference HR was calculated from the PSG ECG signal by applying a QRS peak detection algorithm [35]. Detected R peaks were visually inspected and manually removed or adjusted as needed. After artifact removal, mean overnight values for PSG-based HR and BR were calculated by averaging the HR and BR values across all epochs that were scored. Additionally, HR and BR were characterized for each PSG-defined sleep stage, as described in Appendix A (Table A1).

SOL was defined as the time (in minutes) from lights off to the first epoch scored as any stage other than wake. WASO was computed as the total wake time (in minutes) between sleep onset and sleep offset. TST was computed as the total number of minutes scored as sleep. SE was computed as the ratio between TST and total time in bed (PSG lights off to lights on).

### 2.8. Statistical Analysis

HR and BR analyses were performed using Matlab version R2020 (MathWorks, Natick, MA, USA), and generalized linear model analyses were performed using Matlab version 2019b. All other analyses were performed using Python version 3.8 (Scikit-learn 0.22, NumPy 1.13) and R, version 4.1.1. For each participant, epoch-by-epoch and summary all-night PSG and smart bed measures for HR and BR were matched and compared using the Bland–Altman method. The Bland–Altman method was also used to compare PSG and smart bed values for epoch-by-epoch sleep/wake detection and for all-night summary variables.

Proportional bias and heteroscedasticity were tested visually and using linear regression, as outlined by Menghini et al. [36]. Additionally, the differences in measurement between the smart bed and PSG were tested for normality using the Shapiro–Wilk test.

Bland–Altman [37] plots were generated to assess the level of agreement (LOA) between PSG and equivalent smart bed measures using the guideline proposed by Haghayegh et al. [38]. In the case of negligible proportional bias and lack of heteroscedasticity, comparisons of a single measurement per subject, mean difference (Bias), lower and upper agreement limits (mean difference ± 1.96 × standard deviation [SD]) with their corresponding 95% confidence intervals (CIs) between PSG and smart bed measures were calculated. For repeated measures of epoch-by-epoch analyses, the within- and between-subject variance in measurement difference was obtained and used to compute the bias and LOA along with their corresponding CI [38]. A positive bias indicated that the smart bed platform underestimated the PSG-derived measure, and a negative bias indicated that it overestimated the PSG-derived measure. Pearson correlation coefficients and 95% CI also were calculated.

For epoch-by-epoch sleep/wake detection, Cohen’s kappa, a chance-adjusted measure of agreement [39], was computed for each subject, and the mean and standard deviation were calculated for the entire sample. Because the high imbalance between sleep epochs (74% of all epochs) and wake epochs (26% of all epochs) can result in the underestimation of the true kappa value [39] for overnight profiles, we estimated an adjusted kappa value that accounts for prevalence and bias, as previously described [39]. The following metrics were computed: accuracy (percentage of epochs classified correctly); balanced accuracy (average of the wake and sleep epochs classified correctly); sensitivity (percentage of sleep epochs classified correctly); specificity (percentage of wake epochs classified correctly); precision (proportion of correctly detected sleep epochs among all detected sleep epochs); signal processing d′ (difference in SD between theoretical signal and noise distributions, which is indicative of the model’s ability to detect signals); and AUC.

## 3. Results

### 3.1. Dataset Characteristics

The demographic characteristics of the participants and summary PSG variables are shown in Table 1.

### 3.2. Analysis of Epoch-by-Epoch and All-Night BR and HR

**Figure 3:**
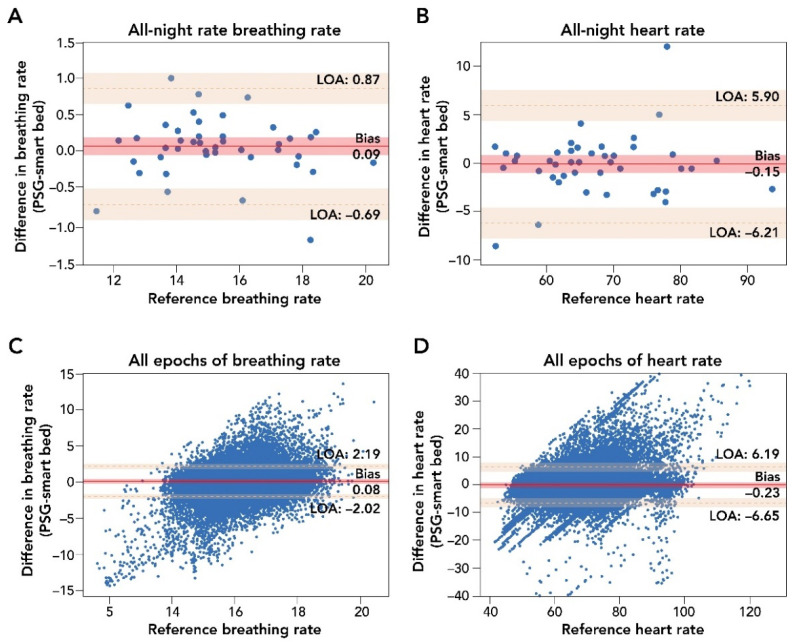
Bland–Altman comparison of all-night and epoch-by-epoch mean BR and HR measurements by smart bed versus PSG. Panels (**A**,**B**): Bland–Altman analysis of mean all-night measurements of BR and HR. The difference in biosignal measurement was plotted as a function of the reference PSG measurement. These analyses indicated a strong agreement of measurements (insignificant bias and narrow LOA) between the smart bed and PSG for both BR and HR. Panels (**C**,**D**): Bland–Altman comparison of epoch-by-epoch BR and HR from the smart bed and PSG. Epoch-by-epoch graphs plot the difference between simultaneously measured values for 30 s epochs from all 45 participants where there were measurements from both systems as a function of the reference (PSG) measurement. In this epoch-by-epoch analysis (minimum of 686 epochs to maximum of 1036 epochs per subject), calculations of bias and LOA were performed as previously described [38]. Epoch-by-epoch analysis indicated a strong agreement on instantaneous biosignals captured by smart bed and PSG. Solid and dotted red lines represent the bias and the upper and lower LOA; shaded areas represent the respective 95% confidence intervals. BR, breathing rate; HR, heart rate; LOA, level of agreement; PSG, polysomnography.

A comparison of smart bed and PSG epoch-by-epoch HR and BR and mean HR and BR measurements during the entire sleep period is presented in Figure 3. Epoch-by-epoch and all-night average differences in HR, as measured by the smart bed and PSG, were tested for the presence of a proportional bias or heteroscedasticity and for normality. No significant dependency was observed, and the differences were normally distributed. After the removal of epochs containing PSG-related artifacts, the smart bed had a coverage rate of 93.6% for HR.

The Bland–Altman analysis for epoch-by-epoch HR (in beats per minute) showed strong agreement between the smart bed and PSG (bias = −0.23 (95% CI: −1.26, 0.78); lower LOA = −6.65 (95% CI: −8.33, −4.97); upper LOA = 6.19 (95% CI: 4.48, 7.85); Figure 3; Table A2) and a strong correlation (r = 0.81 (95% CI: 0.81, 0.82)). The smart bed’s accuracy for detecting HR was comparable to PSG over different sleep stages (see Appendix A). For all-night HR, the Bland–Altman analysis showed a clinically insignificant bias between mean HR from the smart bed versus PSG (bias = −0.15 (95% CI: −1.08, 0.77); lower LOA = −6.21 (95% CI: −7.85, −4.57); upper LOA = 5.90 (95% CI: 4.26, 7.54); Table A2), and a strong correlation was observed between the mean smart bed- and PSG-derived HR (r = 0.94 (95% CI: 0.91, 0.96)).

Epoch-by-epoch and all-night average differences in BR (in breaths per minute) as measured by the smart bed or PSG were similarly tested for the presence of a proportional bias or heteroscedasticity, and for normality. No significant dependency was observed, and the differences were normally distributed. The smart bed had a BR reading for 99.4% of the epochs for which there was also a PSG BR reading (i.e., a 99.4% coverage rate). Comparisons of the smart bed and PSG epoch-by-epoch and all-night average BR measurements in all participants are presented in Figure 3. Bland–Altman comparison of epoch-by-epoch BR between the smart bed and PSG showed insignificant bias, with narrow upper and lower LOAs (bias = 0.08 (95% CI: −0.25, 0.41); lower LOA = −2.02 (95% CI: −2.36, −1.68); upper LOA = 2.19 (95% CI: 1.85, 2.53; Table A3). Likewise, Bland–Altman comparison of all-night BR between the smart bed and PSG showed insignificant bias, with narrow upper and lower LOAs in mean BR (bias = 0.09 (95% CI: −0.03, 0.21); lower LOA = −0.69 (95% CI: −0.90, −0.48); upper LOA = 0.87 (95% CI: 0.66, 1.08; Table A3). Additionally, a strong correlation was observed between the mean smart bed- and PSG-derived BR over the entire night (r = 0.96 (95% CI: 0.94, 0.98)).

### 3.3. Analysis of Epoch-by-Epoch and All-Night Sleep/Wake Detection

**Figure 4:**
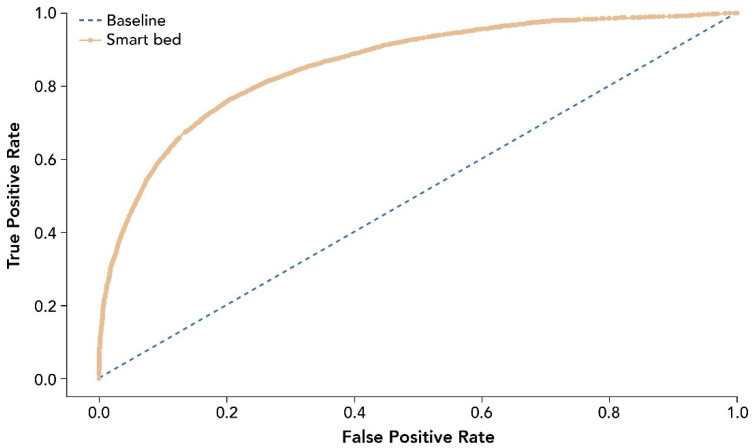
ROC for smart bed prediction of sleep and wake states relative to PSG. The ROC and AUC were assessed as accuracy measures invariant of the prevalence of sleep epochs. The dashed line shows the test with no discriminatory ability (AUC = 0.5). AUC, area under the curve; PSG, polysomnography; ROC, receiver operating characteristic curve.

The entire analysis included 39,855 epochs after data curation and cleaning. Performance measures for the smart bed’s ability to predict sleep and wake states correctly are provided in Table 2. The receiver operating characteristic curve (ROC) for smart bed prediction of sleep and wake states is shown in Figure 4.

### 3.4. Analysis of All-Night Summary Variables

**Figure 5:**
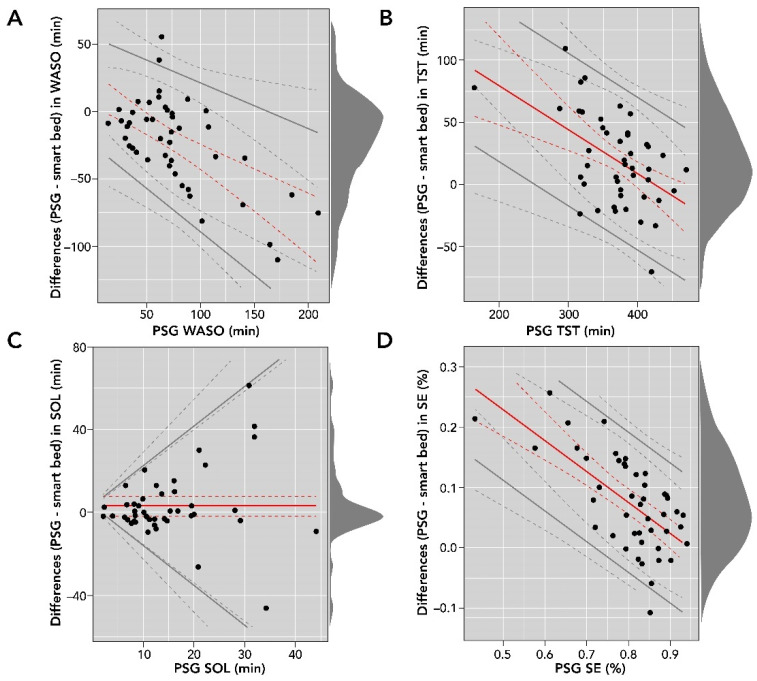
Bland–Altman comparisons of smart bed- versus PSG-derived WASO (**A**); TST (**B**); SOL (**C**); and SE (**D**). The density diagram on the right side of each plot represents the distribution of the differences for each variable. The solid lines are the bias and LOA. The dotted lines represent the corresponding confidence intervals for bias and LOA. LOA, level of agreement; PSG, polysomnography; SE, sleep efficiency; SOL, sleep onset latency; TST, total sleep time; WASO, wake after sleep onset.

Smart bed-collected physiological and motion signals were processed with the DNN to derive sleep/wake epochs and subsequently sleep metrics such as SOL, WASO, TST, and SE. These variables were compared to consensus PSG sleep metrics. A comparison of smart bed and PSG measurements for SOL, WASO, TST, and SE is reported in Table 3, and Bland–Altman analysis plots are shown in Figure 5.

The Bland–Altman analysis of SOL (in minutes) showed proportional bias and heteroscedasticity, where the difference between the measurements increased as the PSG SOL value increased. In addition, the differences were not normally distributed (Shapiro–Wilk test, *p* < 0.05); consequently, they were log-transformed for further analysis. We used basic bootstrapping methods to calculate confidence intervals and linear regression for modeling both the bias and LOAs for plotting and reporting of the Bland–Altman metrics [36]. There was strong agreement between SOL measurements from the smart bed and PSG (bias = 3.0 (95% CI: −1.9 to 7.7); LOAs = bias ± 2.5 [−3.4 + 0.9 × ref]).

The Bland–Altman analysis for WASO (in minutes) showed moderate heteroscedasticity, and the differences were normally distributed. There was moderate agreement between WASO measurements from the smart bed and PSG (bias = 15.28 − 0.49 × ref; LOAs = bias ± 2.46 [16.26 + 0.06 × ref]). Differences in TST (in minutes) were normally distributed and proportional to the size of measurement (r = −0.35 (95% CI: −0.52 to −0.13)). The bias (150.61 − 0.35 × ref) and LOAs (bias ± 61.78) were represented as a function of the size of measurement. Differences in SE (expressed as a percentage) were normally distributed, and the Bland–Altman analysis showed moderate heteroscedasticity and agreement between SE (%) measurements from the smart bed and PSG (bias = 0.48–0.51 × ref; LOAs = bias ± 0.12; Figure 5).

### 3.5. Influence of Demographic and Health Factors on PSG vs. Smart Bed Temporal Concordance

We further examined the role of BMI, sex, age, and AHI on the overall temporal concordance between PSG and smart bed estimations using the coefficient of cross correlation between the corresponding profiles. For HR, the mean ± SD of the cross-correlation was 0.4498 ± 0.2371 (*p* < 0.001), a positive but low correlation. For BR, the mean ± SD of the cross correlation was significantly lower than for HR (0.3629 ± 0.2033; *p* = 0.0312).

In a multiple linear regression with the cross-correlation value for HR as the dependent variable and sex, age, and BMI as covariates, the association with age was not significant (*p* = 0.4628). There was a significant impact of sex, with women having lower concordance between PSG and smart bed estimations (mean ± SE: 0.3945 ± 0.0460 versus 0.5393 ± 0.0507 in men, *p* = 0.0471). Further, increasing BMI was significantly associated with lower cross correlation (*p* = 0.0436). For BR, there were no significant impacts of sex, age, or BMI.

Six of the 45 participants had a negative cross correlation between PSG-based and smart bed-based HR or/and BR, suggestive of substantial discrepancy between temporal variations of PSG versus smart bed signals. Four of these six participants had undiagnosed but clinically significant sleep disorders (obstructive sleep apnea with AHI > 15; periodic leg movement with PLM > 10): subject 6—AHI of 23.9 and PLM of 17.3; subject 11—AHI of 68.9; subject 15—PLM of 46.5; subject 30—PLM of 80.9. This would have excluded them from the study if reported.

## 4. Discussion

We evaluated the performance of the Sleep Number smart bed system in measuring cardiorespiratory endpoints characteristic of sleep and wakefulness, sleep vs. wake status, and summary sleep variables using PSG as the gold standard for comparison. Our dedicated study conformed with multiple recommendations of the guidelines for validation of wearables for sleep outcomes proposed by Depner et al. [20], including recordings obtained under laboratory conditions, manual PSG scoring according to AASM rules, careful synchronization of PSG versus smart bed signals, use of epoch-by-epoch data, use of Bland–Altmann metrics, and use of sensitivity and specificity measures.

The results showed that the measurements of overnight HR and BR generated by the smart bed had biases of <1 beat per minute and <1 breath per minute, respectively. These biases are sufficiently small that they are unlikely to have clinical significance.

With an AUC value of 0.86, the smart bed platform differentiated sleep and wake states reasonably well. The smart bed’s performance was comparable to that of actigraphy found in studies comparing actigraphy to PSG. For example, the smart bed’s accuracy for sleep/wake detection was 0.86 ± 0.11, similar to the accuracy reported for the ActiGraph Link using the Cole-Kripke algorithm (0.891 ± 0.046), the Sadeh algorithm (0.880 ± 0.054), or the Spectrum Plus algorithm (0.904 ± 0.050) [31]. The smart bed’s balanced accuracy was 0.75 ± 0.12, comparable to the ActiGraph Link with Cole-Kripke algorithm (0.752 ± 0.070), ActiGraph Link with Sadeh algorithm (0.779 ± 0.071), and Actiwatch Spectrum Plus algorithm (0.674 ± 0.065) [31]. The kappa value for the smart bed was 0.45 ± 0.17, similar to the kappa values reported for the ActiGraph Link using the Cole–Kripke algorithm (0.482 ± 0.120), the Sadeh algorithm (0.487 ± 0.146), or the Spectrum Plus algorithm (0.424 ± 0.121) [31].

Adjusting for the higher prevalence of sleep compared to wake during overnight measurements, the bias- and prevalence-adjusted kappa value was 0.74 ± 0.11, showing a marked improvement in agreement. Similar to typical actigraphy, due to the prevalence of sleep epochs (of which approximately 74% were sleep in this study), the machine learning model tended to have higher sensitivity for detecting sleep and lower specificity for detecting wake. Specifically, the smart bed’s sensitivity was 0.94 ± 0.05, similar to the sensitivities reported for ActiGraph Link using the Cole–Kripke algorithm (0.936 ± 0.050), the Sadeh algorithm (0.912 ± 0.064), or the Spectrum Plus algorithm (0.982 ± 0.012; [31]). Likewise, its specificity for detecting wake was 0.48 ± 0.18, similar to the specificities reported for ActiGraph Link using the Cole–Kripke algorithm (0.568 ± 0.163), the Sadeh algorithm (0.647 ± 0.163), and the Spectrum Plus algorithm (0.366 ± 0.136) [31].

The performance of the smart bed was also comparable to that of a bed sensor using BCG via pressure-sensitive foil electrodes that detects heartbeat intervals compared with PSG-derived ECG [40,41,42]. For all-night HR, the smart bed’s bias (in beats per minute) was −0.15 (95% CI: −1.08, 0.77), with a lower LOA of −6.21 (95% CI: −7.85, −4.57) and an upper LOA of 5.90 (95% CI: 4.26, 7.54), comparable to the Emfit QS, which had a bias of 0.03 (95% CI: −0.77, 0.83), a lower LOA of −4.37 (95% CI: −5.82, −4.19), and an upper LOA of 4.43 (95% CI: 4.25, 5.88) [8]. Similarly, the smart bed’s bias for all-night BR (in breaths per minute) was 0.09 (95% CI: −0.03, 0.21), with a lower LOA of −0.69 (95% CI: −0.90, −0.48) and an upper LOA of 0.87 (95% CI: 0.66, 1.08). By comparison, the Emfit QS system’s bias for BR was −0.14 (95% CI: −0.57, 0.29), with a lower LOA of −2.49 (95% CI: −2.81, −2.40) and an upper LOA of 2.22 (95% CI: 2.12, 2.53) [8].

All-night-level summary data permit comparison of all-night sleep metrics between the smart bed platform and PSG. Bland–Altman comparisons for an SOL of less than 20 min indicate that the smart bed provided a relatively accurate estimate; however, as SOL increased, the accuracy of detecting SOL decreased. Given that the smart bed does not have direct access to brain activity (which is the gold standard signal to track sleep onset [43]), this is a reasonable level of agreement with PSG.

Bland–Altman comparisons also showed that the smart bed had reasonable accuracy at lower WASOs, but as wake time increased, the platform tended to overestimate WASO. Thus, the smart bed estimated WASO with moderate bias and level of agreement with PSG values. A similar phenomenon was observed for SE; the smart bed was less accurate for estimating SE in studies with lower SE, but its accuracy improved in studies with higher SE. Likewise, the smart bed estimation of sleep was less accurate for studies in which PSG detected shorter sleep durations. Overall, this indicates that the smart bed platform was able to generate a more reliable estimate of TST and other sleep metrics when participants spent less time awake in bed.

In this validation study, we studied a sample of 45 participants who reported having no acute health problems. Higher BMI appeared to negatively influence the accuracy of HR smart bed estimation, which had better concordance with PSG in men than in women. AHI did not significantly influence the accuracy of HR and BR measurements, mainly owing to the small number of patients with AHI > 5.

The validation data presented in this study suggest that the smart bed may provide reliable, longitudinal estimation of sleep quality in an ecologically valid environment, enabling access to sleep metrics in a large population for longer periods of time than is currently possible with PSG [44,45].

The study was limited by a relatively small sample size (*N* = 45) and a single night of simultaneous PSG and smart bed recording in the laboratory. The sample was fairly homogenous in terms of age and degree of adiposity; individuals with previously diagnosed sleep disorders were not included.

Future studies are needed to better characterize the accuracy of the smart bed, with particular regard to longitudinal repeatability and reliability, using data collected over multiple nights for each participant. Further validation is also required to assess the smart bed as a potential screening tool for sleep disorders. Importantly, more research is needed to understand how AHI, periodic leg movement during sleep (PLMS), BMI, and gender influence smart beds’ HR measurements.

## 5. Conclusions

Sleep-dependent changes in cardiorespiratory activity can be reliably detected by the Sleep Number smart bed, and the smart bed platform differentiated between sleep and wake states with performance similar to actigraphy or pressure-sensitive foil electrode bed sensors. All-night summary data analyses indicated that the smart bed provided relatively accurate detection of SOL, WASO, TST, and SE. Future work will build on these results by focusing on sleep-stage detection using the smart bed platform.

## Figures and Tables

**Table 1:** Demographics of the study cohort and PSG-derived summary measures.

Variable	All Participants (*N* = 45)
Male sex, *n* (%)	20 (44.4)
Mean age, years (SD)	41.2 (10.5)
Mean BMI, kg/m^2^ (SD)	25.9 (4.4)
Mean lights out, hh:mm (SD)	22:16 (00:29)
Mean lights on, hh:mm (SD)	05:55 (00:32)
Mean TST, min (SD)	388.7 (46.3)
Mean WASO, min (SD)	54.2 (33.9)
Mean SE, % (SD)	87 (7)
Mean SOL, min (SD)	16.4 (13.6)
Mean BR, bpm * (SD)	14.9 (2.2)
Mean HR, bpm * (SD)	66.4 (9.9)
Mean AHI, events/h (SD)	6.53 (15.1)

AHI, apnea-hypopnea index; BMI, body mass index; bpm, breaths per minute or beats per minute; BR, breathing rate; HR, heart rate; SD, standard deviation; SE, sleep efficiency; SOL, sleep onset latency; TST, total sleep time; WASO, wake after sleep onset. * bpm refers to breaths per minute for BR and beats per minute for HR.

**Table 2:** Performance of the smart bed’s machine learning algorithm for sleep/wake detection.

Performance Measure	Smart Bed Machine Learning Algorithm
AUC	0.86
Accuracy (mean ± SD)	0.86 ± 0.11
Balanced accuracy (mean ± SD)	0.75 ± 0.12
d′ (mean ± SD)	1.47 ± 0.38
Kappa	0.45 ± 0.17
Adjusted kappa (mean ± SD)	0.74 ± 0.11
Precision (mean ± SD)	0.90 ± 0.06
Sensitivity (mean ± SD)	0.94 ± 0.05
Specificity (mean ± SD)	0.48 ± 0.18

AUC, area under the receiver operating curve; SD, standard deviation.

**Table 3:** Comparison of smart bed- and PSG-derived overnight sleep variables.

	SOL (min)	WASO (min)	TST (min)	SE (%)
Smart bed (SD)	13.4 (11.4)	76.8 (43.8)	369.1 (52.7)	0.8 (0.1)
PSG (SD)	16.4 (13.7)	54.2 (33.9)	388.7 (46.4)	0.9 (0.1)
Bias	3.0 (16.8)	15.3 − 0.5 × ref	150.6 − 0.4 × ref	0.5 − 0.5 × ref
Bias CI	[−1.9, 7.7]	b0 = [1.1, 27.6], b1 = [−0.7, −0.3]	b0 = [63.0, 213.9], b1 = [−0.5, −0.1]	b0 = [0.3, 0.6], b1 = [−0.6, −0.3]
Lower LOA	bias − 2.5 (−3.4 + 0.9 × ref)	bias − 2.5 (16.3 + 0.1 × ref)	bias − 61.8	bias − 0.1
Upper LOA	bias + 2.5 (−3.4 + 0.9 × ref)	bias + 2.5 (16.3 + 0.1 × ref)	bias + 61.8	bias + 0.1
LOA CI	c0 = [−22.9, −10.8], c1 = [1.4, 2.2]	c0 = [4.2, 19.4], c1 = [0.0, 0.2]	bias ± [52.8, 74.1]	bias ± [0.1, 0.1]

Sleep metrics were tested for the proportional bias and heteroscedasticity with respect to the reference (PSG) measurement. In the case of proportional bias, the bias coefficients were provided as bias = b0 + b1 × (ref). In the case of heteroscedasticity, the LOA was provided as coefficients of the linear regression model under normality assumption as 95% LOA = bias ± 2.46 (c0 + c1 × (ref)). CI, confidence interval; LOA, level of agreement; PSG, polysomnography; ref, reference measurement; SD, standard deviation; SE, sleep efficiency; SOL, sleep onset latency; TST, total sleep time; WASO; wake after sleep onset.

**Figure A1:**
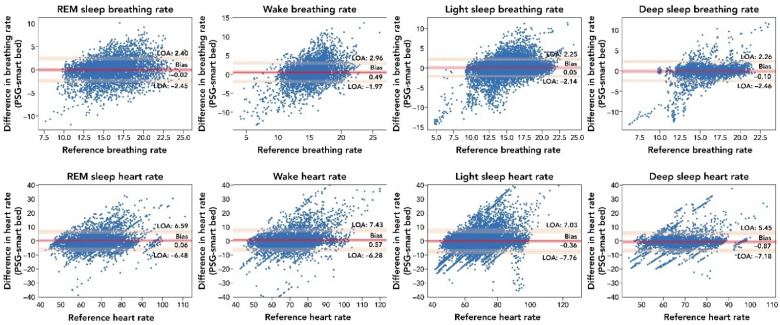
Comparison of the epoch-by-epoch BR and HR measurements by the smart bed versus PSG during different sleep stages. The difference between the smart bed’s versus PSG’s simultaneously measured values for 30 s epochs from all 45 participants was plotted as a function of the reference (PSG) measurement. Consistent insignificant bias and narrow LOA indicated that the smart bed platform was able to track physiological variations during sleep. In these epoch-by-epoch analyses (686–1036 epochs per subject), calculations of bias and LOA were performed as previously described [38]. LOA, level of agreement; PSG, polysomnography; REM, rapid eye movement.

## Data Availability

Data are not publicly available due to limitations in the consent form.

## Appendix A

### Profiles of HR and BR with Regard to Rapid Eye Movement (REM) and Non-REM (NREM) Sleep Stages

We utilized data collected from a 36-year-old woman, with a BMI of 21.6 and AHI of 0.1 (indicating a lack of sleep-disordered breathing) to assess BR and HR during REM and NREM sleep stages. Expected behavior was captured by both PSG and the smart bed. As sleep progressed from onset through the stages of NREM sleep, BR increased slightly and became more regular; these expected physiological changes were detected equally by PSG and smart bed, as shown by the decreased average standard deviation of BR through stages of N1, N2, and N3, irrespective of the method of recording (Table A1). Similar results were seen for HR, where the wake stage had the highest mean and standard deviation in HR relative to all other sleep stages (Table A1).

**Table A1:** Mean epoch-by-epoch HR and BR during different sleep stages. The standard deviation of instantaneous HR and BR were computed on a 15 min, centered, moving window and are grouped and averaged based on PSG sleep stages, representing the 15 min HR and BR variability.

Heart Rate, bpm			Breathing Rate, bpm	
Mean (SD)	Smart Bed	PSG	Smart Bed	PSG
Wake	70.58 (2.52)	71.16 (3.78)	14.56 (1.48)	15.05 (1.10)
Light sleep	67.91 (2.16)	67.11 (2.87)	14.96 (0.98)	15.05 (0.90)
Deep sleep	68.11 (1.42)	67.24 (1.75)	15.76 (0.66)	15.66 (0.75)
REM	67.85 (2.51)	67.89 (2.94)	16.00 (1.34)	15.97 (1.12)
All epochs	67.96 (2.04)	67.72 (2.71)	15.22 (1.04)	15.31 (0.93)

bpm, beats per minute (HR) or breaths per minute (BR); BR, breathing rate; HR, heart rate; PSG, polysomnography; REM, rapid eye movement; SD, standard deviation.

During REM sleep, the breathing pattern was more variable than in NREM, with an overall increase in BR, as expected. REM sleep was characterized by a pronounced increase in HR and HR variability; this increase was captured by both PSG and the smart bed and was consistent with the mean data over the entire population (Table A1). These results suggest an increase in variability in BR and HR during the transition to REM sleep. Furthermore, both methods detected an increase in BR and HR variability through the transition to wake.

The breakdown of the epoch-by-epoch Bland–Altman results for different sleep stages, from lights off to lights on, are illustrated in Figure A1. A minor bias and lower correlation are observed during the wake period, as would be expected due to less accurate measurements of physiological signals expected during wake time due to movements. However, as suggested by the low bias and LOA across different sleep stages (Table A1), the smart bed was able to track BR variations throughout the night.

The breakdown of the epoch-by-epoch Bland–Altman graphs for HR according to the sleep stage from lights out to lights on are provided in Table A2. REM and deep sleep stages have the highest correlation values (r = 0.83 (95% CI: 0.82–0.84) and r = 0.83 (95% CI: 0.83–0.84), respectively), reflecting higher body quiescence during these stages. In contrast, the wake and light-sleep stages had the lowest correlation values (r = 0.79 (95% CI: 0.78–0.80) and r = 0.81 (95% CI: 0.81–0.82), respectively), mostly reflecting movements during the wake and light-sleep stages. Bland–Altman analysis of HR (bpm) across all epochs showed strong agreement between smart bed and PSG (bias = −0.23 (95% CI: −1.26 to 0.78); lower LOA = −6.65 (95% CI: −8.33 to −4.97); upper LOA = 6.19 (95% CI: 4.48–7.85)) and a strong correlation (r = 0.81 (95% CI: 0.81–0.82)).

**Table A2:** Summary of correlation and Bland–Altman metrics for HR for all participants. The number of epochs refers to epochs that were included in the analysis of each group. No coverage refers to the percentages of epochs for which the smart bed did not produce a HR reading, but a PSG HR reading was available. A positive (or negative) bias indicated that the smart bed underestimated (or overestimated) the PSG-derived measure.

Differences			Correlation	
Mean [95% CI]	ΔHR (bpm)	Limits of Agreement (bpm)	r	Number of Epochs (Percent No Coverage)
Over entire night	−0.15 [−1.08, 0.77]	−6.21 [−7.85, −4.57]–5.90 [4.26, 7.54]	0.94 [0.91–0.96]	-
Wake	0.57 [−0.5, 1.65]	−6.28 [−8.04, −4.53]–7.43 [5.68, 9.19]	0.79 [0.78–0.80]	5891 (5.9%)
Light sleep	−0.36 [−1.52, 0.79]	−7.76 [−9.67, −5.86]–7.03 [5.13, 8.94]	0.81 [0.81–0.82]	19,555 (7.1%)
Deep sleep	−0.87 [−1.87, 0.13]	−7.18 [−8.80, −5.56]–5.45 [3.83, 7.07]	0.83 [0.83–0.84]	5870 (0.40%)
REM	0.06 [−0.98, 1.09]	−6.48 [−8.16, −4.8]–6.59 [4.91, 8.28]	0.83 [0.82–0.84]	5934 (7.31%)
All epochs	−0.23 [−1.26, 0.78]	−6.65 [−8.33, −4.97]–6.19 [4.48, 7.85]	0.81 [0.81–0.82]	37,250 (6.40%)

bpm, beats per minute; HR, heart rate; PSG, polysomnography; REM, rapid eye movement.

All-epoch Bland–Altman analysis for BR (bpm) showed a strong agreement between the smart bed and PSG (bias = 0.08) and a positive correlation (r = 0.71; Table A3). The light-sleep and deep-sleep stages had the highest correlation values with BR (r = 0.75 and r = 0.77, respectively), reflecting higher body quiescence (i.e., less motion) during these stages. In contrast, the wake and REM stages had the lowest correlation values (r = 0.5 and r = 0.72, respectively), primarily due to movements during the wake and REM stages.

**Table A3:** Summary of correlation and Bland–Altman metrics for BR for all participants. The number of epochs refers to epochs that were included in the analysis of each group. No coverage refers to the percentages of epochs for which the smart bed did not produce a BR reading, but a PSG BR reading was available. A positive (or negative) bias indicated that the smart bed underestimated (or overestimated) the PSG-derived measure.

Differences			Correlation	
Mean [95% CI]	ΔBR (bpm)	Limits of Agreement (bpm)	r	Number of Epochs (Percent No Coverage)
Over entire night	0.09 [−0.03, 0.21]	−0.69 [−0.90, −0.48]–0.87 [0.66, 1.08]	0.96 [0.94–0.98]	-
Wake	0.49 [0.11, 0.88]	−1.97 [−2.45, −1.49]–2.96 [2.47, 3.44]	0.5 [0.48–0.52]	6238 (1.3%)
Light sleep	0.05 [−0.29, 0.4]	−2.14 [−2.5, −1.78]–2.25 [1.89, 2.61]	0.75 [0.75–0.76]	20,919 (0.6%)
Deep sleep	−0.10 [−0.47, 0.27]	−2.46 [−2.87, −2.05]–2.26 [1.85, 2.67]	0.77 [0.75–0.78]	6105 (0.0%)
REM	−0.02 [−0.41, 0.36]	−2.45 [−2.9, −2.0]–2.4 [1.96, 2.85]	0.72 [0.71–0.73]	6368 (0.0%)
All epochs	0.08 [−0.25, 0.41]	−2.02 [−2.36, −1.68]–2.19 [1.85, 2.53]	0.71 [0.70–0.71]	39,630 (0.6%)

bpm, breaths per minute; BR, breathing rate; PSG, polysomnography; REM, rapid eye movement.

During sleep, the smart bed showed noticeable increases in BR, HR variability, and BR variability in REM sleep, and HR and BR variability decreases in deep NREM. The capability of the smart bed in tracking physiological variations throughout different sleep stages suggest that smart bed technology could be used for detecting sleep stages; this will be the focus of future studies.
